# The transcription factor ATF7 mediates *in vitro* fertilization‐induced gene expression changes in mouse liver

**DOI:** 10.1002/2211-5463.12304

**Published:** 2017-09-11

**Authors:** Yang Liu, Toshio Maekawa, Keisuke Yoshida, Hideki Kaneda, Bruno Chatton, Shigeharu Wakana, Shunsuke Ishii

**Affiliations:** ^1^ Laboratory of Molecular Genetics RIKEN Tsukuba Institute Japan; ^2^ Department of Molecular Genetics and Ph.D. Program in Human Biology School of Integrative and Global Majors University of Tsukuba Japan; ^3^ Technology and Development Team for Mouse Phenotype Analysis RIKEN BRC Tsukuba Japan; ^4^ Université de Strasbourg UMR7242 Biotechnologie et Signalisation Cellulaire Ecole Supérieure de Biotechnologie de Strasbourg Illkirch France

**Keywords:** ATF7, epigenetic change, gene expression change, *in vitro* fertilization, memory, stress

## Abstract

Assisted reproductive technologies, including *in vitro* fertilization (IVF), are now frequently used, and increasing evidence indicates that IVF causes gene expression changes in children and adolescents that increase the risk of metabolic diseases. Although such gene expression changes are thought to be due to IVF‐induced epigenetic changes, the mechanism remains elusive. We tested whether the transcription factor ATF7—which mediates stress‐induced changes in histone H3K9 tri‐ and dimethylation, typical marks of epigenetic silencing—is involved in the IVF‐induced gene expression changes. IVF up‐ and downregulated the expression of 688 and 204 genes, respectively, in the liver of 3‐week‐old wild‐type (WT) mice, whereas 87% and 68% of these were not changed, respectively, by IVF in ATF7‐deficient (*Atf7* ^*−/−*^) mice. The genes, which are involved in metabolism, such as pyrimidine and purine metabolism, were upregulated in WT mice, but not in *Atf7* ^*−/−*^ mice. Of the genes whose expression was upregulated by IVF in WT mice, 37% were also upregulated by a loss of ATF7. These results indicate that ATF7 is a key factor in establishing the memory of IVF effects on metabolic pathways.

**Abbreviations**
ATF: activating transcription factor
IVF: *in vitro* fertilization

Assisted reproductive technologies (ARTs), including *in vitro* fertilization (IVF), are increasingly used for infertility treatment in humans. More than 6 million children have been conceived through IVF and other ARTs 1. Although ARTs are thought to be safe, multiple studies suggest that children and adolescents conceived by IVF are at increased risk of metabolic diseases, due to elevated blood pressure, fasting glucose, and peripheral body fat 2, 3. In particular, young adults conceived by IVF display reduced insulin sensitivity and may be more susceptible to the deleterious metabolic consequence of obesogenic environment 4. Animal studies further confirm the influence of IVF on metabolism at later developmental stages. IVF alters the composition of lipid in mouse fetal liver, and adult mice conceived by IVF display increased fasting glucose levels and impaired glucose tolerance 4, 5. The metabolome in adult fat and liver is also altered by IVF, and metabolites enriched in steroidogenesis and pyrimidine metabolism are elevated by IVF 6.

Accumulating evidence in the Developmental Origins of Health and Disease (DOHaD) field supports the idea that stressful events in early development stages increase susceptibility to chronic diseases in later life 7. The IVF procedure exposes the gametes and embryo to an environment that dramatically deviates from natural conception. Manipulation and culture *in vitro* of gametes and early embryo during IVF would introduce several external stimuli, such as high oxygen, pH, and temperature fluctuations, and mechanical stress, which induces stress‐activated responses of gametes and embryo 8, 9, 10. The preimplantation embryo is particularly vulnerable to environment disturbances, and these stressful factors could alter the development trajectory of the embryo, leading to long‐term effects on gene expression patterns at later stages 7, 11. IVF induces gene expression pattern changes in blastocysts, placenta, and adult tissues, including skeletal muscle, fat, liver, and islets 12, 13, 14.

Epigenetic regulation of gene expression is mainly mediated by DNA methylation, post‐translational histone modifications, and noncoding RNA. Substantial research indicates that IVF increases the risk of imprinting diseases, such as Beckwith–Wiedemann syndrome, by affecting epigenetic reprogramming and resulting in alteration of gene expression 15, 16. Children conceived by IVF exhibit altered DNA methylation at imprinted genes, such as *MEST* and *H19/IGF2* 11, 17, 18. IVF also induces the dysregulation of microRNA in mouse embryos, particularly the downregulation of miR‐199a‐5p, which is involved in the regulation of glucose metabolism 19. Furthermore, IVF alters histone modification at the promoter region of the gene encoding thioredoxin‐interacting protein, which plays an important role in glucose and redox hemostasis, in blastocysts, and in adult adipose tissues 14. However, the molecular mechanism underlying the lasting effects of IVF on alteration of metabolic homeostasis through epigenetic reprogramming remains elusive.

The ATF2 subfamily of transcription factors belongs to the ATF/CREB superfamily whose members bind to the cAMP response element. It consists of three members, ATF2 (originally named CRE‐BP1) 20, 21, ATF7 (originally named ATF‐a) 22, and CRE‐BPa 23, all of which contain a phosphorylation site for the stress‐activated protein kinase p38 24, 25. In the absence of stress, ATF7 silences gene transcription by promoting the formation of heterochromatin‐like structures via recruitment of the histone H3K9 trimethyltransferase ESET or the histone H3K9 dimethyltransferase G9a 26, 27. Psychological stress and pathogen infection stimulate p38‐mediated phosphorylation of ATF7 and its subsequent release from target gene promoters, leading to a decrease in H3K9me3 or H3K9me2 and elevated gene expression 26, 27. The disrupted heterochromatin‐like structure is not completely recovered, and the higher level of basal expression is maintained for a long period 27. On the other hand, both ATF2 and CRE‐BPa act as transcriptional activators by recruiting coactivator CBP 28. Drosophila ATF2 (dATF2), the ATF7 homolog, has a similar function to ATF7 and also contributes to the formation of heterochromatin. During early embryogenesis, heat shock stress induces dATF2 phosphorylation and disruption of heterochromatin, and this disrupted heterochromatin can be inherited by the next generation, implying that dATF2 is involved in stress‐induced epigenetic changes in germ cells and early embryos 29. Based on these results, we speculated that ATF7 may also play a role in stress‐induced epigenetic changes in gametes and early embryos during the IVF process and may contribute to gene expression changes at later stages.

Here, we investigated the liver transcriptome in 3‐week‐old mice generated by IVF and normal mating. We found that IVF induced expression changes in nearly 900 genes, many of which were involved in metabolic pathways. Interestingly, the effect of IVF on metabolic genes was reduced in the absence of ATF7. The effect of ATF7 deletion and IVF on the expression of metabolic genes was to some extent similar. Our data imply that ATF7 contributes to IVF‐induced gene expression changes by mediating stress‐induced epigenetic changes.

## Materials and methods

### Mice and IVF

ATF7‐deficient (*Atf7* ^*−/−*^) (C57BL6/JJmsSlc background) mice were generated as described previously 26. Using *Atf7* ^*−/−*^ male and female (C57BL6/JJmsSlc background) or wild‐type (WT) male and female (C57BL6/JJmsSlc background) mice, *Atf7* ^*−/−*^ or WT mice were generated by IVF or natural mating. IVF was performed as follows. Sperm were collected from the caudae epididymides of male mice and allowed to diffuse in human tubal fluid (HTF) fertilization medium 30. After preincubation for approximately 1 h to allow for capacitation, the sperm were used for insemination. Meanwhile, female mice were superovulated using intraperitoneal injections of 7.5I U PMSG and HCG (Serotropin and Gonatropin; ASKA Pharmaceutical Co., Tokyo, Japan) with an interval of 48 h between injections. Approximately 15–17 h after the HCG injection, the oocyte–cumulus complexes were collected from the oviducts of superovulated female mice. Then, the complexes from several female mice were placed in fertilization medium. Insemination was performed by adding the preincubated sperm suspension to the fertilization HTF medium (Table S1) containing complexes and cultured at 37 °C with 5% CO_2_ in air. Twenty‐four hours after insemination, two‐cell embryos were transferred into the oviducts of pseudopregnant ICR females (CLEA Japan, Tokyo, Japan) mated to vasectomized ICR males. Recipient mice were transplanted 12 embryos into each oviduct (i.e., a total of 24 embryos per recipient). All pups were delivered naturally after embryo transfer. Nine and 12 recipients were transferred, and pups were born from 7 and 12 recipients for WT and *Atf7* ^*−/−*^ mice, respectively (Table S1). Although the IVF group had a tendency of slightly higher litter size, this difference may not be critical to compare between WT and *Atf7* ^*−/−*^ pups, because such tendency was also found in either WT or *Atf7* ^*−/−*^ pups. Experiments were conducted in accordance with the guidelines of the Animal Care and Use Committee of the RIKEN Institute.

### Gene expression analysis using array

The liver samples were isolated from 3‐week‐old male mice derived from natural mating or IVF. Three livers were obtained from offspring from different recipient mothers (IVF groups) or from pregnant mice (natural mating groups), and used for gene expression analysis. The data of body weight of these 3‐week‐old mice, when the livers were prepared, indicated that the IVF group of WT mice had a tendency of lower body weight compared to the natural mating group of WT mice (Fig. S1). However, there was no difference between the IVF and natural mating group of *Atf7* ^*−/−*^ mice (see Discussion). Total RNA was extracted using TRIzol (Invitrogen, Carlsbad, CA, USA) based on the manufacturer's protocol. Single‐strand cDNA was prepared with WT Expression Kit (Ambion, Invitrogen Life Technologies, Carlsbad, CA, USA) and then labeled using the WT terminal labeling kit (Ambion) according to the manufacturer's manual. Samples were analyzed by microarray using the Mouse Gene 1.0 ST Array (Affymetrix Inc., Santa Clara, CA, USA). The raw (CEL) data were normalized using the RMA method in the r package *affy*. The comparisons of gene expression were implemented using the linear models for microarray data (Limma) package 31, in which *P*‐value was adjusted using Benjamini and Hochberg's method, one kind of post hoc tests. The differentially expressed genes (DEGs) were defined with an adjusted *P*‐value of ≤0.05 and an absolute log_2_ fold‐change (log_2_FC) of ≥0.6. The pathways enrichment analysis for DEGs was conducted using the Kyoto Encyclopedia of Genes and Genomes (KEGG) database 32. Hierarchical clustering analysis was performed using the log_2_ signal intensity of IVF‐induced DEGs in WT mice.

### Quantitative RT‐PCR

Reverse transcription PCR was performed using OneStep SYBR Green PCR mix (Takara, Shiga, Japan) following the manufacturer's instructions. The qRT‐PCR was performed using a 7500 Fast Real‐time PCR System (Applied Biosystems, Foster City, CA, USA). Primers GGCTCCTCTATGATGGCCG and AAGCCTTTCTGAACAGCCAGC were used for *Mest*; primers AACGGTGGAGATGGATTCCA GATG and GACTTGCTGCAGAGAACTTGATCC were used for *Nt5e*; primers TCAGTGTACCATGATTGCCTTG and GAACCTGCTCTGCCTGTTG were used for *Idi1*; primers GCTCCAAGCAGATGCAGCA and CCGGATGTGAGGCAGCAG were used for *36B4*; and primers GGGTGTCCTCCCTGGAAAAG and TCAGCT GAGCCACCTCATTG were used for *Atf7*. The reference gene *36B4* was used as a relative control, and data were analyzed using the 2^−ΔΔCt^ method 33.

### Western blotting

Protein extracts were prepared from liver samples by homogenization in radioimmunoprecipitation buffer (50 mm Tris/HCl pH 8.0, 150 mm sodium chloride, 0.1% SDS, 0.5% sodium deoxycholate, 1.0% NP‐40, and protease inhibitors). Proteins were separated on a 7.5% SDS/PAGE and then transferred to PVDF membrane. After treatment with the anti‐ATF7 monoclonal (1A7) antibody or anti‐α‐tubulin antibody (Abcam, ab4074) at 4 °C overnight, the blots were incubated with a peroxidase‐conjugated anti‐mouse IgG1 (Santa Cruz Biotechnology, Santa Cruz, CA, USA) or anti‐rabbit (Invitrogen) secondary antibody followed by ECL detection (GE Healthcare UK Limited, Buckinghamshire, UK) according to the manufacturer's instructions.

## Results

### Effect of IVF on gene expression profiles in liver

**Figure 1:**
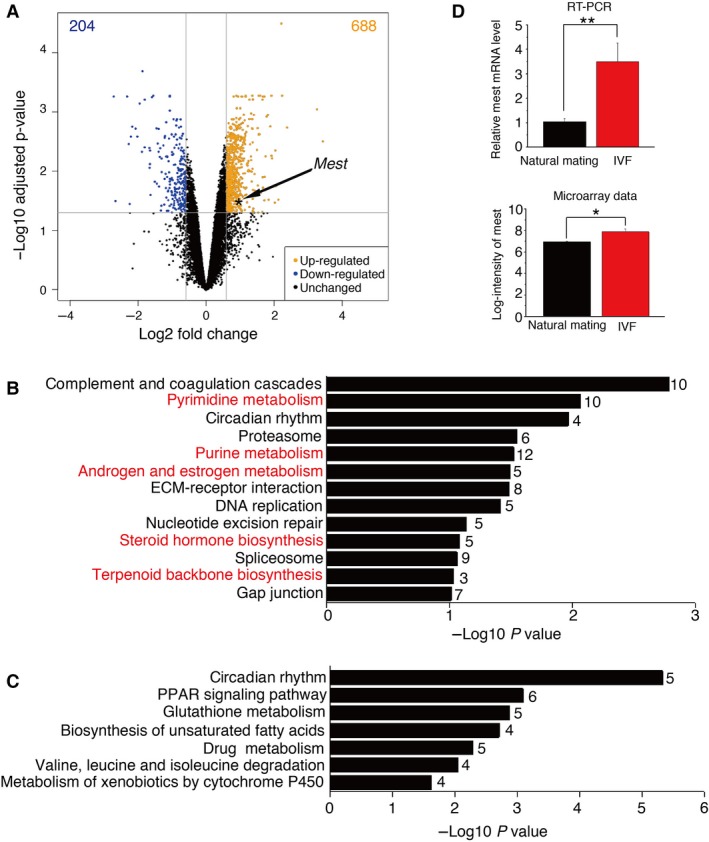
Effects of IVF on gene expression pattern in liver. (A) Volcano plot for the analysis of differential gene expression between IVF and natural mating samples. IVF upregulated 688 genes (yellow spots) and downregulated 204 genes (blue spots). The data point for *Mest* is marked with an asterisk. (B and C) KEGG pathway enrichment analysis was performed for genes up‐ and downregulated by IVF, respectively. *P*‐values for the gene enrichment analysis were calculated by a modified Fisher's exact test, and the number to the right of each bar is the number of genes involved in each pathway. Thirteen KEGG pathways (*P* < 0.1) were overrepresented among upregulated genes, and five of these were associated with metabolism (in red) (B). Seven KEGG pathways (*P* < 0.1) were overrepresented among downregulated genes (C). (D) Gene expression of *Mest* in natural mating and IVF samples (*n* = 3) analyzed by qRT‐PCR and microarray, respectively. Bars are means + SEM. *P*‐value for qRT‐PCR data: (paired Student's *t*‐test) ***, < 0.001. Adjusted *P*‐value for microarray expression data: *, < 0.05. Note that the y‐axis for qRT‐PCR and microarray indicates relative level and log intensity, respectively.

To explore the gene expression pattern changes induced by IVF, we investigated the liver transcriptome in 3‐week‐old male mice generated by either IVF or normal mating (control), using an array covering the mouse whole‐genome transcript with 750 000 unique oligonucleotide probes. This analysis identified 688 genes upregulated and 204 genes downregulated in IVF liver compared with the control (Fig. 1A and Data S1). The original microarray data are retrieved from Gene Expression Omnibus (GEO) database (GEO number: GSE99859). To investigate the pathways associated with DEGs, the gene lists of up‐ and downregulated genes were analyzed for KEGG pathway enrichment using the DAVID database. Thirteen pathways were overrepresented among the upregulated genes, including complement and coagulation cascades, gap junction, and ECM–receptor interaction. Several metabolism‐associated pathways were identified, such as pyrimidine metabolism, purine metabolism, and steroid hormone biosynthesis (Fig. 1B and Data S4). These results are consistent with the report that metabolites enriched in pyrimidine metabolism and steroidogenesis are elevated by IVF 6. The downregulated genes were also enriched for genes in metabolic‐related pathways, including PPAR signaling, valine, leucine, and isoleucine degradation, and biosynthesis of unsaturated fatty acids (Fig. 1C and Data S4), implying that IVF mainly disturbs metabolic processes in liver. The circadian rhythm pathway was overrepresented among both up‐ and downregulated genes. The imprinted gene *Mest* was expressed more highly in IVF liver than in control liver (log_2_FC = 0.92, adjusted *P*‐value = 0.03). To verify the microarray data, we examined the relative expression level of *Mest* by qRT‐PCR using *36B4* as an internal control. The result was consistent with the microarray data and indicated that the expression levels of *Mest* were significantly higher in livers of mice conceived by IVF than in livers of control mice (Fig. 1D). As reported previously 11, 17, 18, the expression level of another imprinted gene *Igf2* was upregulated by IVF (log_2_FC = 0.588, adjusted *P*‐value = 0.031), whereas the expression level of *H19* was not affected (log_2_FC = 0.462, adjusted *P*‐value = 0.742).

### The influence of IVF on gene expression is reduced in Atf7^*−/−*^ mice

**Figure 2:**
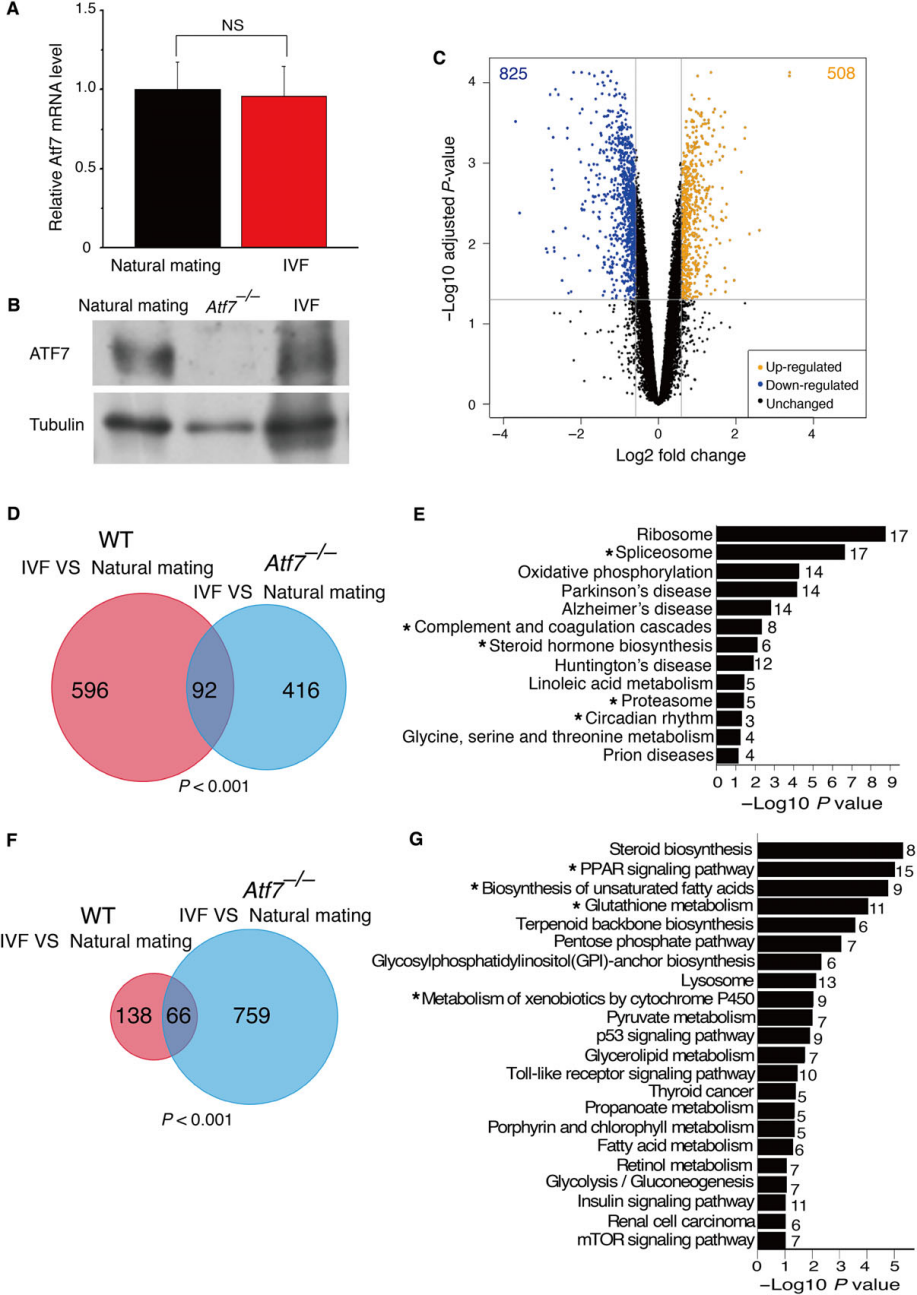
Ablation of ATF7 reduces the effects of IVF on gene expression in liver. (A) The levels of *Atf7* mRNA in liver of naturally and IVF‐derived mice. Bars are means + SEM. *P*‐value: (paired Student's *t*‐test) NS, > 0.05. (B) ATF7 protein levels in liver of naturally and IVF‐derived mice were examined by western blotting. *Atf7* ^*−/−*^ mice were used as a negative control, and tubulin was the loading control. (C) Volcano plot for the analysis of differential gene expression between *Atf7* ^*−/−*^ IVF and *Atf7* ^*−/−*^ natural mating samples. IVF upregulated 508 genes (yellow spots) and downregulated 825 genes (blue spots). (D) Venn diagram showing overlap in IVF‐induced upregulated genes between WT and *Atf7* ^*−/−*^ livers. Statistical significance of the overlap was examined by Fisher's exact test. (E) KEGG pathway enrichment analysis for IVF‐induced upregulated genes in *Atf7* ^*−/−*^ mice. *P*‐values for the gene enrichment analysis were calculated by a modified Fisher's exact test, and the number to the right of each bar is the number of genes involved in each pathway. Thirteen KEGG pathways (*P* < 0.1) were overrepresented among upregulated genes, and five pathways were common with those overrepresented among IVF‐induced upregulated genes in WT mice (marked by asterisks). (F) Venn diagram showing overlap in IVF‐induced downregulated genes between WT and *Atf7* ^*−/−*^ livers. Statistical significance of the overlap was examined by Fisher's exact test. (G) KEGG pathway enrichment analysis for IVF‐induced downregulated genes in *Atf7* ^*−/−*^ mice. *P*‐values for the gene enrichment analysis were calculated by a modified Fisher's exact test, and the number to the right of each bar is the number of genes involved in each pathway. Twenty‐two KEGG pathways (*P* < 0.1) were overrepresented among downregulated genes, and four pathways were common with those overrepresented among IVF‐induced downregulated genes in WT mice (marked by asterisks).

To explore whether ATF7 is involved in IVF‐induced gene expression changes, we first tested the effect of IVF on ATF7 expression levels in liver. There was no difference in *Atf7* mRNA levels between IVF and control livers (Fig. 2A). Using liver from *Atf7* ^*−/−*^ mice as a negative control, western blotting also showed that the level of ATF7 protein in IVF livers is comparable to that in control livers (Fig. 2B). Therefore, IVF did not affect the expression of ATF7 in liver.

Next, we explored whether the absence of ATF7 affected the influence of IVF by comparing the liver gene expression profiles between IVF‐conceived *Atf7* ^*−/−*^ mice and naturally conceived *Atf7* ^*−/−*^ mice (*Atf7* ^*−/−*^ control). In *Atf7* ^*−/−*^ liver, 508 genes were upregulated and 825 genes were downregulated by IVF (Fig. 2C and Data S2). Comparison of the IVF‐induced genes in WT (688 genes) with those in *Atf7* ^*−/−*^ (508 genes) liver indicated that the expression of 87% (596/688 genes) of the IVF‐induced genes in WT was not affected by IVF in *Atf7* ^*−/−*^ mice (Fig. 2D). Only 13% (92/688 genes, *P* < 0.001) were induced by IVF in both WT and *Atf7* ^*−/−*^ mice, and these included genes associated with the circadian rhythm (i.e., *NPAS2*,* ARNTL*, and *CRY1*) and complement and coagulation cascades (i.e., *MBL1*,* F13B*, and *FGA*). The KEGG pathway analysis showed that the IVF‐induced genes in *Atf7* ^*−/−*^ liver were enriched for genes in 13 pathways (Fig. 2E and Data S5). Among them, only five pathways were common with those identified for upregulated genes in WT mice: circadian rhythm, spliceosome, complement and coagulation cascades, proteasome, and steroid hormone biosynthesis. Other pathways identified in WT mice, including purine metabolism and terpenoid backbone biosynthesis, were not observed in *Atf7* ^*−/−*^ mice.

Comparison of the IVF‐induced downregulated genes in WT (204 genes) with those in *Atf7* ^*−/−*^ (825 genes) liver indicated that the expression of 68% (138/204 genes) of the IVF‐induced downregulated genes in WT was not affected by IVF in *Atf7* ^*−/−*^ mice (Fig. 2F). About 32% (66/204 genes, *P* < 0.001) were downregulated by IVF in both WT and *Atf7* ^*−/−*^ mice, and these included genes associated with the PPAR signaling pathway (i.e., *FADS2* and *PLTP*) and glutathione metabolism (i.e., *GSTT2, GSTP1*, and *OPLAH*). The KEGG pathway analysis showed that the IVF‐induced downregulated genes in *Atf7* ^*−/−*^ liver were enriched for genes in 22 pathways (Fig. 2G and Data S5). Among them, four pathways were common with those identified for downregulated genes in WT mice: PPAR signaling pathway, biosynthesis of unsaturated fatty acids, glutathione metabolism, and metabolism of xenobiotics by cytochrome P450. Other pathways identified in WT mice, including circadian rhythm, were not observed in *Atf7* ^*−/−*^ mice.

These results indicate that a loss of ATF7 inhibits the considerable percentage of IVF‐induced gene expression change, and this was more evident in the IVF‐induced upregulated genes than in the downregulated genes. Our previous studies showed that ATF7 silences gene expression via recruitment of the histone H3K9 trimethyltransferase ESET or the histone H3K9 dimethyltransferase G9a 26, 27. In response to stress, ATF7 is phosphorylated and released from the target genes, which causes upregulation of the target genes. Thus, the upregulated genes by IVF or ATF7 mutation may be the key to understand the effect of IVF. Downregulation of the genes by IVF or ATF7 mutation is likely to be induced indirectly.

### ATF7 ablation can partially mimic the effect of IVF

**Figure 3:**
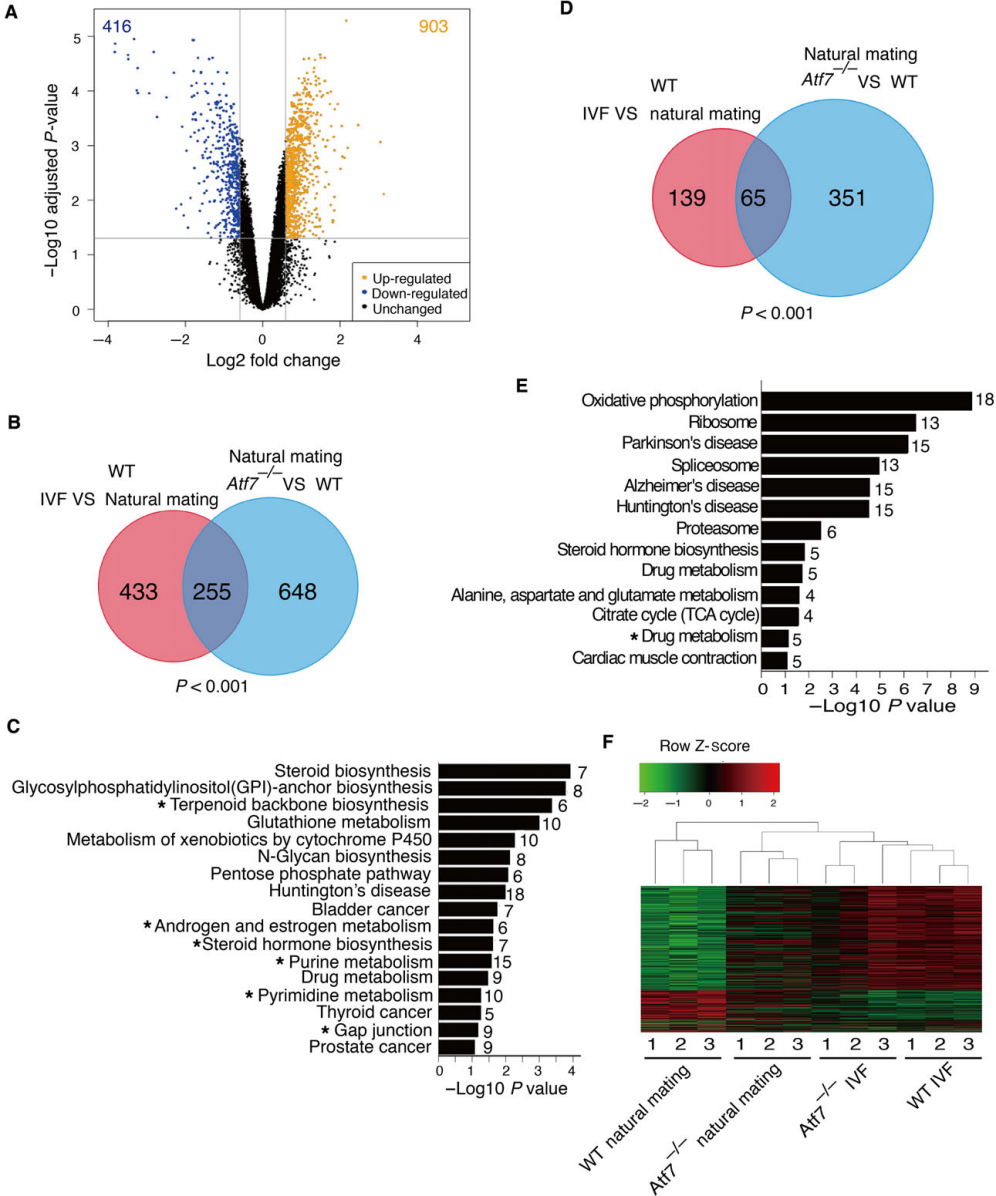
ATF7 deficiency partially mimics the effects of IVF on gene expression in liver. (A) Volcano plot for the analysis of differential gene expression between *Atf7* ^*−/−*^ and WT samples. Loss of ATF7 resulted in upregulation of 903 genes (yellow spots) and downregulation of 416 genes (blue spots). (B) Venn diagram showing overlap between IVF‐induced and ATF7 deficiency‐induced upregulated genes. Statistical significance of the overlap was examined by Fisher's exact test. (C) KEGG pathway enrichment analysis for ATF7 deficiency‐induced upregulated genes. *P*‐values for the gene enrichment analysis were calculated by a modified Fisher's exact test, and the number to the right of each bar is the number of genes involved in each pathway. Seventeen KEGG pathways (*P* < 0.1) were overrepresented among upregulated genes, and six pathways were common with those overrepresented among IVF‐induced upregulated genes in WT mice (marked by asterisks). (D) Venn diagram showing overlap between IVF‐induced and ATF7 deficiency‐induced downregulated genes. Statistical significance of the overlap was examined by Fisher's exact test. (E) KEGG pathway enrichment analysis for ATF7 deficiency‐induced downregulated genes. *P*‐values for the gene enrichment analysis were calculated by a modified Fisher's exact test, and the number to the right of each bar is the number of genes involved in each pathway. Thirteen KEGG pathways (*P* < 0.1) were overrepresented among downregulated genes, and one pathway was common with those overrepresented among IVF‐induced downregulated genes in WT mice (marked by asterisks). (F) Unsupervised hierarchical clustering using IVF‐induced differential gene expression between IVF and natural mating samples. Red and green indicate higher and lower expression levels, respectively.

ATF7 acts as a transcriptional repressor and mediates stress‐induced gene expression. Therefore, *Atf7* ^*−/−*^ mice exhibit some similar phenotypes to WT mice exposed to stress 26, 27. Thus, we compared the gene expression profiles of *Atf7* ^*−/−*^ and WT livers. In *Atf7* ^*−/−*^ liver, 903 genes were upregulated and 416 genes were downregulated compared with WT liver (Fig. 3A and Data S3). Comparison of the 903 upregulated genes with the 688 IVF‐induced genes showed that 37% (255/688 genes) of the IVF‐induced genes also exhibited higher expression levels in *Atf7* ^*−/−*^ than in WT mice (Fig. 3B). The 903 upregulated genes were enriched for genes in 17 pathways (Fig. 3C). The majority of these pathways were closely related to metabolism, including purine and pyrimidine metabolism, steroid biosynthesis, and steroid hormone biosynthesis, implying that ATF7 plays an important role in the regulation of metabolism in liver. Interestingly, six pathways enriched for the IVF‐induced genes were common to those enriched for the ATF7 ablation‐induced genes, including purine metabolism, pyrimidine metabolism, and terpenoid backbone biosynthesis (Fig. 3C and Data S6).

Comparison of the 416 downregulated genes with the 204 IVF‐induced downregulated genes showed that 32% (65/204 genes) of the IVF‐induced genes also exhibited lower expression levels in *Atf7* ^*−/−*^ than in WT mice (Fig. 3D). The 416 downregulated genes were enriched for genes in 13 pathways, which contained not only the metabolism‐related pathways but also other pathways (Fig. 3E and Data S6). Only the drug metabolism pathway was common to those enriched for the ATF7 ablation‐induced downregulated genes.

Hierarchical clustering analysis allows us to visualize the relationship between the different experiment components. By using DEGs identified in IVF versus control, all 12 samples were divided into two major clusters. The three WT control samples formed a cluster, and *Atf7* ^*−/−*^ samples from naturally conceived mice grouped together with IVF samples (Fig. 3F). These data further demonstrate the potential role of ATF7 in mediating the IVF‐induced gene expression changes, particularly for genes associated with metabolic pathways.

### Gene expression changes in metabolic pathways

**Figure 4:**
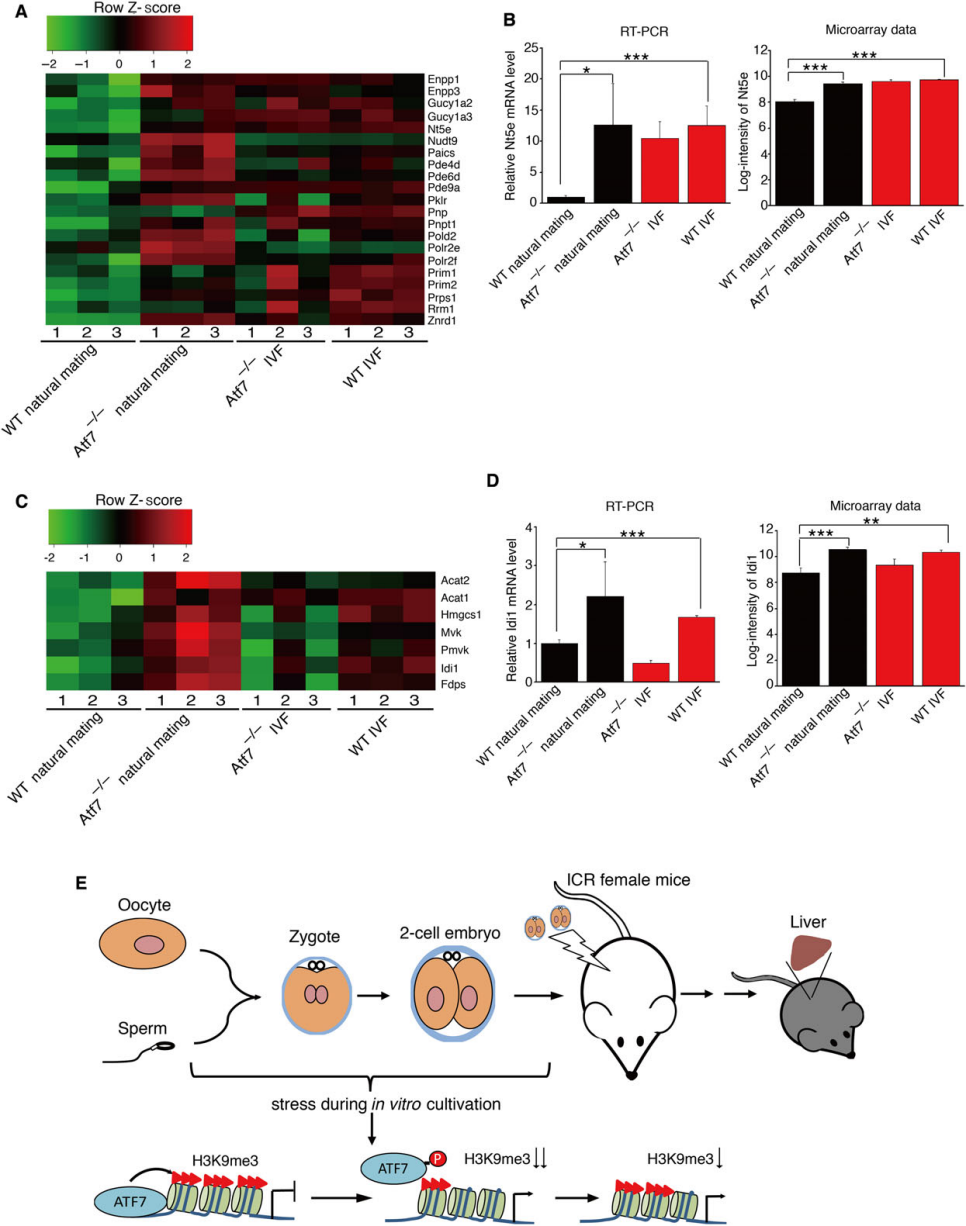
Involvement of ATF7 in the effect of IVF on metabolic pathways. (A) Heat map for expression profile of genes involved in purine metabolism. Red and green indicate higher and lower expression levels, respectively. (B) The expression levels of *Nt5e* in four groups of samples (*n* = 3) analyzed by qRT‐PCR and microarray, respectively. Bars are means + SEM. *P*‐value for qRT‐PCR: (paired Student's *t*‐test) *, < 0.05, ***, < 0.001. Adjusted *P*‐value for microarray expression data: ***, < 0.001. Note that the y‐axis for qRT‐PCR and microarray indicates relative level and log intensity, respectively. (C) Heat map for expression profile of genes involved in terpenoid backbone biosynthesis. Red and green indicate higher and lower expression levels, respectively. (D) The expression levels of *Idi1* in four groups of samples (*n* = 3) analyzed by qRT‐PCR and microarray, respectively. Bars are means + SEM *P*‐value for qRT‐PCR: (paired Student's *t*‐test) *, < 0.05, ***, < 0.001. Adjusted *P*‐value for microarray expression data: **, < 0.01, ***, < 0.001. (E) The potential role of ATF7 in IVF‐induced gene expression changes.

Dozens of genes induced by IVF and involved in metabolic pathways were also upregulated in the *Atf7* ^*−/−*^ control, whereas their expression levels were not increased by IVF in *Atf7* ^*−/−*^ mice. This observation led us to postulate that ATF7 may mainly contribute to IVF‐induced upregulation of genes in metabolic pathways. Hence, we checked the expression profiles of metabolic genes involved in two pathways: purine metabolism and terpenoid backbone biosynthesis. The heat map of expression profiles of genes involved in purine metabolism showed that the majority of genes exhibited higher expression levels in *Atf7* ^*−/−*^ control, *Atf7* ^*−/−*^ IVF, and WT IVF groups than in the WT control (Fig. 4A). This tendency was confirmed by the measurement of *Nt5e* mRNA levels using qRT‐PCR. *Nt5e* mRNA levels were 10‐fold higher in *Atf7* ^*−/−*^ control, *Atf7* ^*−/−*^ IVF, and WT IVF groups than in the WT control (Fig. 4B). However, genes involved in terpenoid backbone biosynthesis were only upregulated in *Atf7* ^*−/−*^ control and WT IVF groups. Gene expression levels in *Atf7* ^*−/−*^ IVF were not higher than in the WT control (Fig. 4C). Results of qRT‐PCR showed that the *Idi1* mRNA level was upregulated by IVF and ATF7 ablation, but decreased in *Atf7* ^*−/−*^ IVF (Fig. 4D). Expression levels of the transcriptional activator *Srebf2*, which is a key player in lipid metabolism, were higher in the *Atf7* ^*−/−*^ control than in the WT control (log_2_FC = 0.754762, adjusted *P*‐value = 0.0004) (Data S3). In the absence of ATF7, IVF led to a reduction in *Srebf2* expression level (log_2_FC = −0.68, adjusted *P*‐value = 0.0009). The expression level of *Srebf2* may explain the expression patterns of genes involved in terpenoid backbone biosynthesis and indicates that ATF7 contributes to IVF‐induced gene expression changes synergistically with other factors.

## Discussion

This study indicates that ATF7 is involved in the memory of IVF‐induced gene expression pattern changes in the liver. Our previous study showed that ATF7 silences target genes by forming a heterochromatin‐like structure via recruitment of histone H3K9 trimethyltransferase ESET/SETDB1 26 or histone H3K9 dimethyltransferase G9a 27, and that certain stress induces ATF7 phosphorylation by p38, leading to ATF7 release. This causes a decrease in H3K9me3 or H3K9me2 and subsequent transcriptional induction. Once the heterochromatin‐like structure is disrupted by stress, it is not completely recovered and the partially disrupted structure and the higher basal expression level are retained for a long period. A similar scenario may occur in the memory of IVF‐induced gene expression changes (Fig. 4E). Note that IVF may induce the phosphorylation of ATF7 and its release, but the ATF7 mRNA and protein levels are not affected by IVF. It is important whether IVF induces phosphorylation of ATF7, but its analysis is technically difficult at present. We have previously shown that phosphorylation of ATF7 is only transiently induced by stress 26, 27, which is a characteristic of the p38‐induced phosphorylation. Therefore, two‐cell embryos have to be used to test the IVF‐induced phosphorylation of ATF7. The antibodies to detect phosphorylated ATF7 (P‐ATF7), which are now available, also react with phosphorylated ATF2 (P‐ATF2), because the amino acid sequences around the p38 phosphorylation sites are similar between ATF7 and ATF2. Thus, western blotting analysis is required to distinguish between P‐ATF7 and P‐ATF2, and immunostaining analysis cannot be used. However, many two‐cell embryos (10^4^–10^5^) are needed to detect P‐ATF7 by western blotting, which is almost impossible. However, based on multiple reports, we speculate that IVF induces phosphorylation of ATF7. During IVF, especially *in vitro* culture of zygotes and two‐cell embryos, the level of reactive oxygen species (ROS) is elevated 34. A high level of intracellular glutathione, which reduces ROS, is important for bovine embryo development after *in vitro* maturation 35. As ROS induces p38 activation 36, an increased ROS level during *in vitro* culture of zygotes and two‐cell embryos leads to ATF7 phosphorylation. According to the Database of Transcriptome in Mouse Early Embryos (http://dbtmee.hgc.jp/gene_card.php?id=2002900), ATF7 is expressed in oocytes, zygotes, and two‐cell embryos at least at low levels. ATF7 phosphorylation and its release from target genes lead to a decrease in H3K9me3 and/or H3K9me2, which is not completely recovered. Thus, a partly disrupted heterochromatin‐like structure and high basal expression of some ATF7 target genes may be maintained during the development of liver at least until 3 weeks of age. Although IVF may affect the body weight of newborn mice and postnatal growth curve, we did not measure them. Instead, the body weight at 3 weeks after birth was measured when the livers were prepared (Fig. S1). Although the IVF group of WT mice had a tendency of lower body weight compared to the natural mating group of WT mice (but not significant), there was no difference between the IVF and natural mating group of *Atf7* ^*−/−*^ mice. We previously reported that the body weight of *Atf7* ^*−/−*^ mice was slightly lower than that of wild‐type mice 37. These results appear to be consistent with the hypothesis that IVF affects the body weight by changing histone H3K9 methylation via ATF7.

Metabolism‐related genes are overrepresented among the IVF‐induced genes in 3‐week‐old liver. These consist of two types of genes, whose expression is directly or indirectly regulated by ATF7. Why does IVF induce the memory of upregulation of metabolism‐related genes? This could be an adaptation to the IVF stress. When zygotes and early embryos are cultured *in vitro*, cells must adapt to the nonphysiological extracellular stimulus via modulation of metabolism, such as the concentration of some metabolites. For example, to combat the higher ROS level during *in vitro* culture, more antioxidants may be produced. The genes involved in complement and coagulation cascades are also upregulated by IVF. Complement is a key system for homeostasis 38, and so those genes may play a role in maintaining homeostasis after receiving IVF stress. These changes may benefit cells when they are exposed to IVF stress, but may induce long‐term harmful consequences at later stages after transfer to female recipient mice.

One idea is that if ATF7 is a key mediator in determining the phenotype of IVF, IVF in *Atf7* ^*−/−*^ mice should result in mice having a phenotype and gene expression similar to naturally mated *Atf7* ^*−/−*^ mice. In other words, the removal of ATF should nullify the effects of IVF. In WT liver, IVF induced up‐ and downregulation of 892 genes, and 82% (734/892 genes) of them were not affected by IVF in *Atf7* ^*−/−*^ mice (Fig. S2). On the other hand, in *Atf7* ^*−/−*^ liver, IVF induced the up‐ and downregulation of 1333 genes, and 88% of them (1175/1333 genes) were not induced by IVF in WT liver. Thus, IVF induced the expression change of different sets of genes in WT and *Atf7* ^*−/−*^ mice. This suggests that ATF7 is a key mediator for the IVF‐induced memory of expression change of specific set of genes in WT mice and that another pathway involving other factor(s) such as microRNA 19 may work to mediate the IVF‐induced memory of expression change of different set of genes in *Atf7* ^*−/−*^ mice (Fig. S2). Activation of another minor pathway in the absence of the major pathway is often observed in various biological phenomena such as immune system. Thus, our results suggest the presence of some backup system, which acts to induce the memory of the IVF effect in the absence of ATF7. ATF7 is not the only inducer of epigenetic change in response to stress. Two substrates of the Krebs cycle, fumarate and succinate, are competitive inhibitors of multiple α‐ketoglutarate‐dependent dioxygenases, including histone demethylases and the TET family of 5‐methylcytosine hydroxylases involved in DNA demethylation 39. Therefore, a change in metabolism induced by IVF may cause epigenetic change via those metabolites in the absence of ATF7, although it remains unknown how inhibition of histone demethylases and DNA demethylation affects the epigenetic status of specific genes.

In *Atf7* ^*−/−*^ liver, IVF did not induce the memory of upregulation of genes involved in purine metabolism, whose expression levels were higher than in WT liver. This suggests that ATF7 suppresses transcription of those genes by forming a heterochromatin‐like structure and that loss of ATF7 or IVF‐induced release of ATF7 upregulated their expression. On the other hand, although the expression of genes in terpenoid backbone biosynthesis was also upregulated by a loss of ATF7, IVF oppositely suppressed their expression in *Atf7* ^*−/−*^ liver. This might be caused by inducing some transcriptional repressor by IVF in *Atf7* ^*−/−*^ liver. As described above, some metabolites such as substrates of the Krebs cycle change the epigenetic states of some genes. Such regulation may induce a specific repressor for the genes involved in terpenoid backbone biosynthesis, but not for the genes involved in purine metabolism. It might be interesting to think about the relationship between specific metabolism pathways and adaptation to IVF stress.

Feuer *et al*. 6 previously demonstrated that IVF exaggerated male–female differences in metabolite concentration in the mouse liver, with the majority of changes affecting amino acid and lipid metabolites. As we analyzed the gene expression patterns in the liver of only male mice, it remains unknown whether the IVF‐induced and ATF7‐dependent changes in gene expression occur in both males and females. However, the major change observed in our study was upregulation of genes involved in purine metabolism. The IVF‐induced change affecting purine metabolites were detected in both males and females in the study by Feuer *et al*. 6, suggesting that the IVF‐induced and ATF7‐dependent changes in gene expression may occur in both males and females. We analyzed the liver of 3‐week‐old mice, while the liver of 29‐week‐old mice was analyzed by Feuer *et al*. 6. In both studies, some metabolic pathways, such as purine metabolism, changed by IVF, suggesting that at least some gene expression change observed in 3‐week‐old liver might maintain until the adult stage.

This study indicates that ATF7 is a major regulator in inducing the memory of IVF effects. At present, it is difficult to identify ATF7‐binding genes in zygotes and two‐cell embryos due to technical limitations. However, in the future, identification of those ATF7 target genes and measurement of the IVF‐induced epigenetic changes at those genes will be useful to predict the effect of IVF in adulthood.

## Data accessibility

The original microarray data have been deposited in the Gene Expression Omnibus (GEO) database (GEO number: GSE99859).

## Author contributions

YL, TM, KY, HK, SW, and SI conceived and designed the experiments. YL, TM, and HK performed the experiments. BC provided the monoclonal antibodies. YL, TM, KY, and SI analyzed the data. YL, SW, and SI wrote the manuscript.

## Supporting information


**Fig. S1.** Body weight of 4 groups of mice at 3 weeks after birth (*n* = 3). *P*‐value (paired Student's *t*‐test): *, < 0.05; **, < 0.01; NS, not significant.

**Fig. S2.** In WT liver, IVF induced up‐ and down‐regulation of 892 genes, and 82% (734/892 genes) of them were not affected by IVF in *Atf7* ^*−/−*^ mice.

**Table S1.** Number of embryos transferred and litter size.

**Data S1.** Up‐ and down‐regulated genes by IVF in WT mice (for Fig. 1A).
**Data S2.** Up‐ and down‐regulated genes by IVF in Atf7 mutant mice (for Fig. 2C).
**Data S3.** Up‐ and down‐regulated genes by ATF7 deficiency (for Fig. 3A).
**Data S4.** The KEGG pathway analysis for up‐and down‐regulated genes by IVF in WT mice (for Fig. 1B,C).
**Data S5.** The KEGG pathway analysis for up‐ and down‐regulated genes by IVF in Atf7 mutant mice (for Fig. 2E,G).
**Data S6.** The KEGG pathway analysis for up‐ and down‐regulated genes by ATF7 deficiency (for Fig. 3C,E).
