# Supplementary figures and images for: The transcription factor ATF7 mediates in vitro fertilization‐induced gene expression changes in mouse liver
Source: FEBS Open Bio. 2017 Sep 11;7(10):1598–610. doi: 10.1002/2211-5463.12304 (PMC5623699; doi:10.1002/2211-5463.12304)

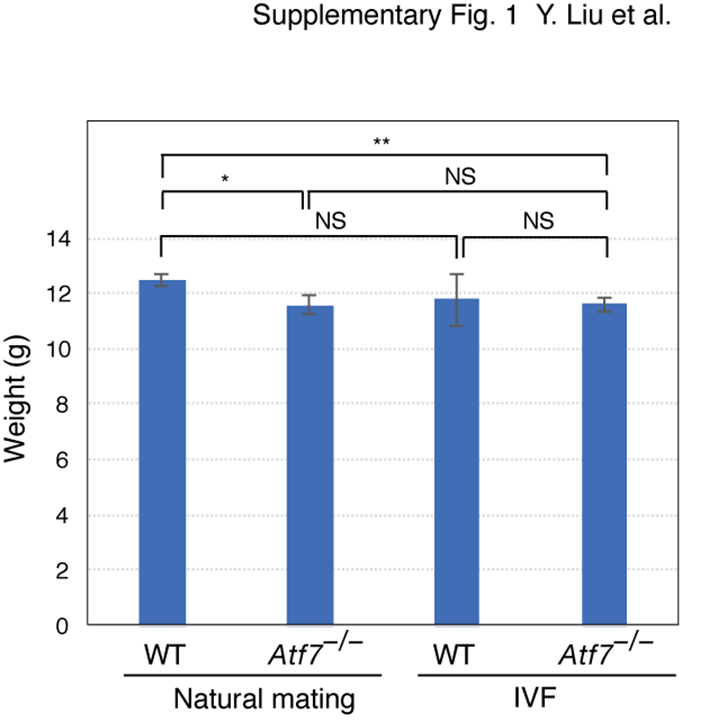

Supplement: Supplementary file 1 — Fig. S1. Body weight of 4 groups of mice at 3 weeks after birth (n = 3). P‐value (paired Student's t‐test): *, < 0.05; **, < 0.01; NS, not significant. [file FEB4-7-1598-s001.tif]

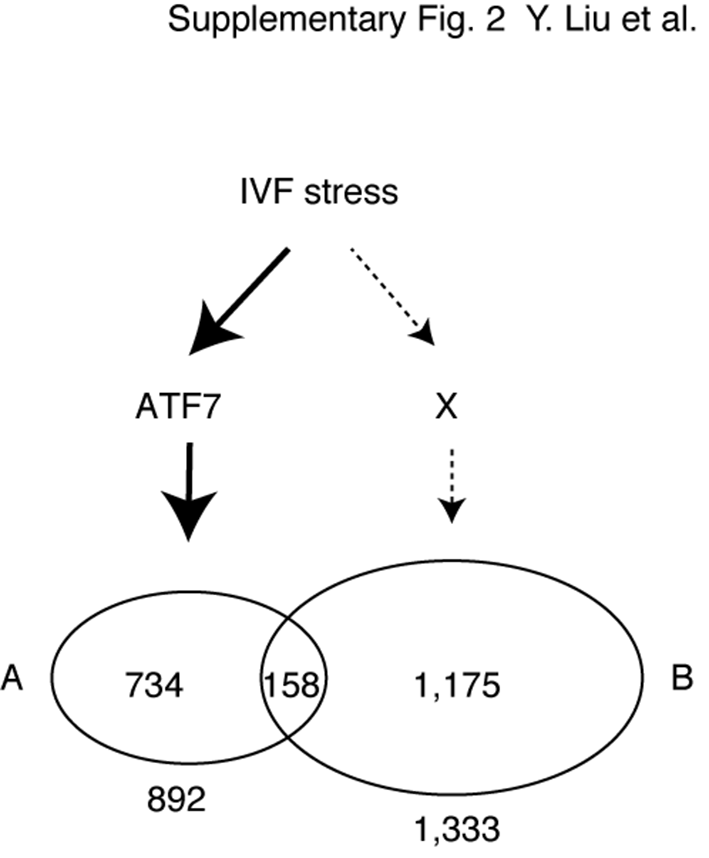

Supplement: Supplementary file 2 — Fig. S2. In WT liver, IVF induced up‐ and down‐regulation of 892 genes, and 82% (734/892 genes) of them were not affected by IVF in Atf7 −/− mice. [file FEB4-7-1598-s002.tif]
