# Supplementary material for: The transcription factor ATF7 mediates in vitro fertilization‐induced gene expression changes in mouse liver
Source: FEBS Open Bio. 2017 Sep 11;7(10):1598–610. doi: 10.1002/2211-5463.12304 (PMC5623699; doi:10.1002/2211-5463.12304)
Supplement: Supplementary file 3 — Table S1. Number of embryos transferred and litter size. [file FEB4-7-1598-s003.docx]

**Supplementary Table 1** Number of embryos transferred and litter size.

| Group | # of recipient or pregnant mice | Number of embryos transferred | Litter size |
| --- | --- | --- | --- |
| IVF  WT | 1 | 24 | 13 |
|  | 2 | 24 | 7 * |
|  | 3 | 24 | 7 * |
|  | 4 | 24 | 11 |
|  | 5 | 24 | 15 |
|  | 6 | 24 | 8 * |
|  | 7 | 24 | 0 |
|  | 8 | 24 | 0 |
|  | 9 | 24 | 0 |
| IVF  *Atf7^—/—^* | 1 | 24 | 6 * |
|  | 2 | 24 | 6 * |
|  | 3 | 24 | 11 |
|  | 4 | 24 | 12 |
|  | 5 | 24 | 11 |
|  | 6 | 24 | 11 |
|  | 7 | 24 | 13 |
|  | 8 | 24 | 13 |
|  | 9 | 24 | 10 |
|  | 10 | 24 | 13 |
|  | 11 | 24 | 5 * |
|  | 12 | 24 | 9 |
| Natural mating  WT | 1 | NA (not applicable) | 6 * |
|  | 2 | NA | 6 * |
|  | 3 | NA | 7 * |
| Natural mating  *Atf7^—/—^* | 1 | NA | 6 * |
|  | 2 | NA | 7 * |
|  | 3 | NA | 7 * |

* Each one mouse from the group indicated by asterisk was used to prepare liver.
