# Supplementary material for: The transcription factor ATF7 mediates in vitro fertilization‐induced gene expression changes in mouse liver
Source: FEBS Open Bio. 2017 Sep 11;7(10):1598–610. doi: 10.1002/2211-5463.12304 (PMC5623699; doi:10.1002/2211-5463.12304)
Supplement: Supplementary file 4 — Data S1. Up‐ and down‐regulated genes by IVF in WT mice (for Fig. 1A). Data S2. Up‐ and down‐regulated genes by IVF in Atf7 mutant mice (for Fig. 2C). Data S3. Up‐ and down‐regulated genes by ATF7 deficiency (for Fig. 3A). Data S4. The KEGG pathway analysis for up‐and down‐regulated genes by IVF in WT mice (for Fig. 1B,C). Data S5. The KEGG pathway analysis for up‐ and down‐regulated genes by IVF in Atf7 mutant mice (for Fig. 2E,G). Data S6. The KEGG pathway analysis for up‐ and down‐regulated genes by ATF7 deficiency (for Fig. 3C,E). [file FEB4-7-1598-s004.docx]

Data S1. Up- and down-regulated genes by IVF in WT mice (for Fig.  1A).
Data S2. Up- and down-regulated genes by IVF in Atf7 mutant mice (for Fig.  2C).
Data S3. Up- and down-regulated genes by ATF7 deficiency (for Fig. 3A).
Data S4. The KEGG pathway analysis for up-and down-regulated genes by IVF in WT mice (for Fig. 1BC).
Data S5. The KEGG pathway analysis for up- and down-regulated genes by IVF in Atf7 mutant mice (for Fig. 2E,G).
Data S6. The KEGG pathway analysis for up- and down-regulated genes by ATF7 deficiency (for Fig. 3C,E).
